# Supplementary material for: Temporal profiling of redox-dependent heterogeneity in single cells
Source: eLife. 2018 Jun 5;7:e37623. doi: 10.7554/eLife.37623 (PMC6023615; doi:10.7554/eLife.37623)
Supplement: Supplementary file 1. [file elife-37623-supp1.docx]

**Supplementary File 1.** Comparison of OxD values of wild type and knockout strains (related to Figure 2F).

| Strain | cytosol | peroxisome |
| --- | --- | --- |
| *Δpex3* | 9.0E-01 | < 1.0E-05 |
| *Δcat2* | 2.5E-01 | 1.0E-03 |
| *Δatg36* | 1.6E-01 | < 1.0E-05 |
| *Δahp1* | 4.0E-01 | 1.0E-03 |

The p-values for T-student test calculated using two tail similar variance.
