# Supplementary material for: Temporal profiling of redox-dependent heterogeneity in single cells
Source: eLife. 2018 Jun 5;7:e37623. doi: 10.7554/eLife.37623 (PMC6023615; doi:10.7554/eLife.37623)
Supplement: Supplementary file 5. [file elife-37623-supp5.docx]

#### Supplementary File 5. Comparison of wild type and knockout strains OxD values (related to Figure 6E).

| Strain | Day 1 | Day 2 | Day 3 |
| --- | --- | --- | --- |
| *Δtsa2* | 1.27E-02 | 1.34E-04 | 2.60E-01 |
| *Δdhh1* | 1.36E-05 | 6.73E-08 | 1.86E-08 |
| *Δhbt1* | 5.76E-04 | 3.38E-05 | 5.04E-06 |
| *Δpnc1* | 3.50E-03 | 3.67E-05 | 3.05E-06 |
| *Δhsp30* | 4.51E-07 | 8.78E-08 | 8.32E-05 |
| *Δgdh2* | 6.37E-01 | 1.07E-05 | 4.63E-06 |

The p-values for T-student test calculated using two tail similar variance.
