# Supplementary material for: Temporal profiling of redox-dependent heterogeneity in single cells
Source: eLife. 2018 Jun 5;7:e37623. doi: 10.7554/eLife.37623 (PMC6023615; doi:10.7554/eLife.37623)
Supplement: Supplementary file 12. [file elife-37623-supp12.docx]

#### Supplementary File 12. Comparison of wild type and knockout strains OxD values (related to Figure 7).

| Strain | Day 1 | Day2 |
| --- | --- | --- |
| *Δhsp30* | 4.51E-07 | 8.78E-08 |
| *Δsse2* | 1.85E-02 | 2.62E-02 |
| *Δyro2* | 3.75E-03 | 1.91E-03 |
| *Δhsp78* | 1.10E-02 | 2.52E-03 |

The p-values for T-student test calculated using two tail similar variance
